# Benzoic acid–2-{(*E*)-[(*E*)-2-(2-pyridyl­methyl­idene)hydrazin-1-yl­idene]meth­yl}pyridine (2/1)

**DOI:** 10.1107/S1600536810040651

**Published:** 2010-10-20

**Authors:** Hadi D. Arman, Trupta Kaulgud, Edward R. T. Tiekink

**Affiliations:** a Department of Chemistry, The University of Texas at San Antonio, One UTSA Circle, San Antonio, Texas 78249-0698, USA; b Department of Chemistry, University of Malaya, 50603 Kuala Lumpur, Malaysia

## Abstract

The asymmetric unit of the title cocrystal, C_12_H_10_N_4_·2C_7_H_6_O_2_, comprises a single mol­ecule of benzoic acid and one half-mol­ecule of 2-pyridine­aldazine situated about a centre of inversion. The carboxyl group is coplanar with the benzene ring to which it is connected [O—C—C—C = −172.47 (12)°] and similarly, the 2-pyridine­aldazine mol­ecule is planar (r.m.s. deviation of the 16 non-H atoms = 0.017 Å). In the crystal, mol­ecules are connected into a non-planar three-mol­ecule aggregate [dihedral angle between the benzene and pyridyl ring connected by the hydrogen bond = 61.30 (7)°] with a twisted Z-shape. Layers of 2-pyridine­aldazine mol­ecules in the *ab* plane are sandwiched by benzoic acid mol­ecules being connected by O—H⋯N and C—H⋯O inter­actions, the latter involving the carbonyl O atom so that each benzoic acid mol­ecule links three different 2-pyridine­aldazine mol­ecules. Inter­digitated layers stack along the *c* axis.

## Related literature

For related studies on co-crystal formation involving the isomeric *n*-pyridine­aldazines, see: Broker *et al.* (2008 ▶); Arman *et al.* (2010*a* ▶,*b* ▶).
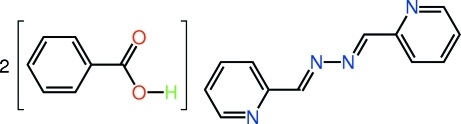


## Experimental

### 

#### Crystal data

C_12_H_10_N_4_·2C_7_H_6_O_2_
*M* *_r_* = 454.48
Triclinic, 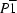

*a* = 4.4509 (7) Å
*b* = 11.3635 (17) Å
*c* = 12.0612 (17) Å
α = 108.985 (6)°
β = 99.830 (9)°
γ = 97.849 (10)°
*V* = 556.16 (14) Å^3^
*Z* = 1
Mo *K*α radiation
μ = 0.09 mm^−1^
*T* = 98 K
0.40 × 0.29 × 0.12 mm

#### Data collection

Rigaku AFC12/SATURN724 diffractometer
Absorption correction: multi-scan (*ABSCOR*; Higashi, 1995 ▶) *T*_min_ = 0.759, *T*_max_ = 1.000
2790 measured reflections
1935 independent reflections
1811 reflections with *I* > 2σ(*I*)
*R*_int_ = 0.022

#### Refinement

*R*[*F* ^2^ > 2σ(*F* ^2^)] = 0.042
*wR*(*F* ^2^) = 0.117
*S* = 1.00
1935 reflections
158 parameters
1 restraint
H atoms treated by a mixture of independent and constrained refinement
Δρ_max_ = 0.20 e Å^−3^
Δρ_min_ = −0.23 e Å^−3^

### 

Data collection: *CrystalClear* (Molecular Structure Corporation & Rigaku, 2005 ▶); cell refinement: *CrystalClear*; data reduction: *CrystalClear*; program(s) used to solve structure: *SHELXS97* (Sheldrick, 2008 ▶); program(s) used to refine structure: *SHELXL97* (Sheldrick, 2008 ▶); molecular graphics: *ORTEP-3* (Farrugia, 1997 ▶) and *DIAMOND* (Brandenburg, 2006 ▶); software used to prepare material for publication: *publCIF* (Westrip, 2010 ▶).

## Supplementary Material

Crystal structure: contains datablocks global, I. DOI: 10.1107/S1600536810040651/hg2725sup1.cif

Structure factors: contains datablocks I. DOI: 10.1107/S1600536810040651/hg2725Isup2.hkl

Additional supplementary materials:  crystallographic information; 3D view; checkCIF report

## Figures and Tables

**Table 1:** Hydrogen-bond geometry (Å, °)

*D*—H⋯*A*	*D*—H	H⋯*A*	*D*⋯*A*	*D*—H⋯*A*
O1—H1*o*⋯N1	0.85 (2)	1.88 (2)	2.7269 (16)	177 (2)
C11—H11⋯O2^i^	0.95	2.54	3.1811 (19)	125
C12—H12⋯O2^ii^	0.95	2.59	3.4647 (19)	154

Symmetry codes: (i) 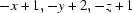; (ii) 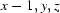.

**Fig. 1.**
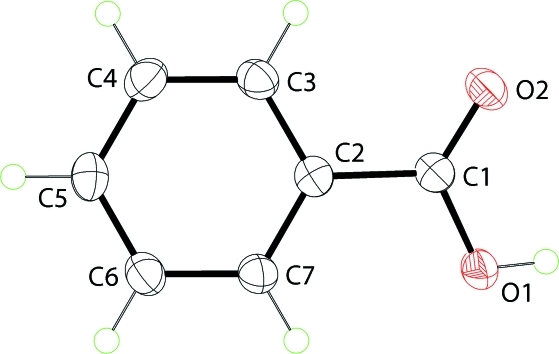
Molecular structure of benzoic acid found in the structure of (I) showing atom-labelling scheme and displacement ellipsoids at the 50% probability level

**Fig. 2.**
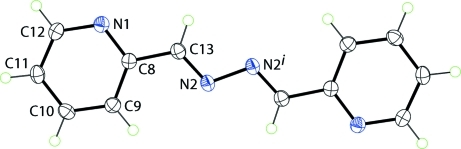
Molecular structure of 2-pyridinealdazine found in the structure of (I) showing atom-labelling scheme and displacement ellipsoids at the 50% probability level. The molecule is disposed about a centre of inversion and i = 2 - x, 1 - y, 1 - z.

**Fig. 3.**
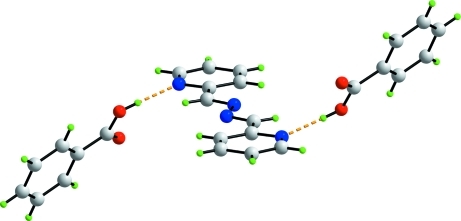
The three molecule aggregate in (I) highlighting the extended chair conformation. The O—H···N hydrogen bonds are shown as orange dashed lines.

**Fig. 4.**
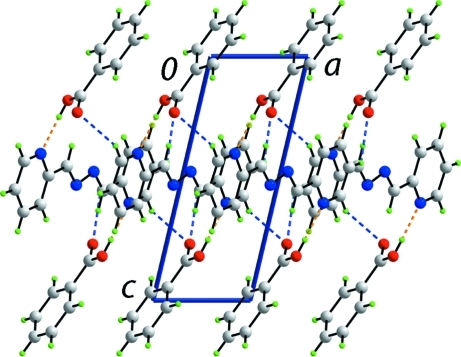
A view of the supramolecular layer in (I) whereby 2-pyridinealdazine molecules are sandwiched by benzoic acid molecules. The O—H···N hydrogen bonds and C—H···O contacts are shown as orange and blue dashed lines, respectively.

**Fig. 5.**
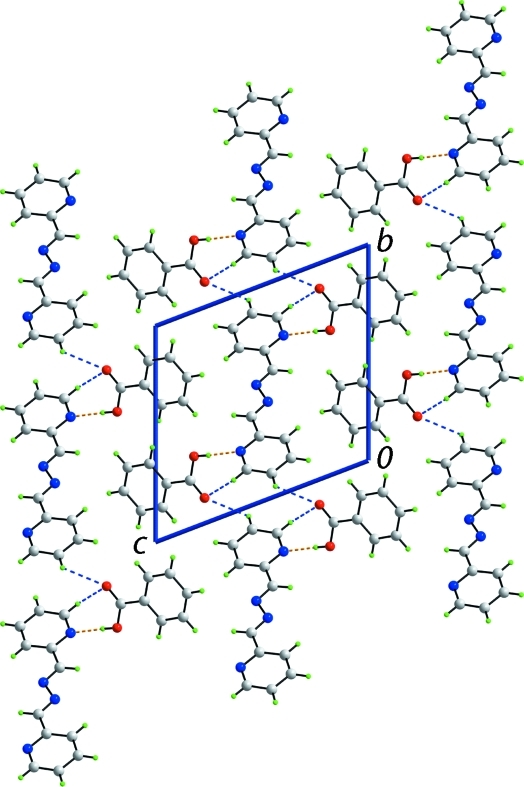
A view in projection down the a axis showing the stacking of layers comprising three molecule aggregates along c. The O—H···N hydrogen bonds and C—H···O contacts are shown as orange and blue dashed lines, respectively.

## supplementary crystallographic information

### Comment

Co-crystallization experiments with the isomeric n-pyridinealdazines have led to the characterization of several co-crystals (Broker *et al.*, 2008; Arman *et al.*, 2010*a*; Arman *et al.*, 2010*b*), and in continuation of these studies, the co-crystallization of benzoic acid and 2-pyridinealdazine was investigated. This lead to the isolation of the title 2/1 co-crystal, (I).

The asymmetric unit in (I) comprises a molecule of benzoic acid, Fig. 1, and half a molecule of 2-pyridinealdazine, with the latter disposed about a centre of inversion, Fig. 2. The constituents of (I) are connected by O—H···N hydrogen bonds, Table 1, to generate a centrosymmetric three molecule aggregate, Fig. 3. The benzoic acid molecule is planar as seen in the value of the O1—C1—C2—C3 torsion angle of -172.47 (12) °. Similarly, the 2-pyridinealdazine molecule is planar with the r.m.s. deviation of the 16 non-hydrogen atoms being 0.017 Å. However, the three molecule aggregate is not planar as the benzene ring forms a dihedral angle of 61.30 (7) ° with the pyridyl ring to which it is hydrogen bonded. Overall, when viewed normal to the plane through 2-pyridinealdazine, the aggregate has the shape of a twisted letter *Z*.

In the crystal packing, the 2-pyridinealdazine molecules pack in the *ab* plane with the benzoic acid molecules sandwiching these, Fig. 4. The connections are mediated by the aforementioned O—H···N hydrogen bond as well as C—H···O interactions formed by the carbonyl-O atom; each benzoic acid molecule links three distinct 2-pyridinealdazine molecules. Inter-digitated layers stack along the *c* axis.

### Experimental

Yellow crystals of (I) were isolated from the 2/1 co-crystallization of benzoic acid (Sigma Aldrich, 0.24 mmol) and 2-[(*E*)-[(*E*)-2-(pyridin-2-ylmethylidene)hydrazin-1-ylidene]methyl]pyridine (Sigma Aldrich, 0.12 mmol) in ethanol, m. pt. 351–353 K.

IR assignment (cm^-1^): 2600 (br) ν(O—H); 1691 ν(C=O); 1627 ν(C═N); 1469, 1451, 1416 ν(C–C(aromatic)); 1627 ν(C—N); 777 δ(C—H).

### Refinement

C-bound H-atoms were placed in calculated positions (C–H 0.95–0.99 Å) and were included in the refinement in the riding model approximation with *U*_iso_(H) set to 1.2*U*_eq_(C). The O-bound H-atom was located in a difference Fourier map and was refined with a distance restraint of O–H 0.84±0.01 Å, and with *U*_iso_(H) = 1.5*U*_eq_(O). In the final refinement a low angle reflection evidently effected by the beam stop was omitted, *i.e*. (0 1 1).

### Crystal data

**Table:**

C_12_H_10_N_4_·2C_7_H_6_O_2_	*Z* = 1
*M**_r_* = 454.48	*F*(000) = 238
Triclinic, *P*1	*D*_x_ = 1.357 Mg m^−^^3^
Hall symbol: -P 1	Mo *K*α radiation, λ = 0.71073 Å
*a* = 4.4509 (7) Å	Cell parameters from 2619 reflections
*b* = 11.3635 (17) Å	θ = 2.1–40.2°
*c* = 12.0612 (17) Å	µ = 0.09 mm^−^^1^
α = 108.985 (6)°	*T* = 98 K
β = 99.830 (9)°	Block, yellow
γ = 97.849 (10)°	0.40 × 0.29 × 0.12 mm
*V* = 556.16 (14) Å^3^	

### Data collection

**Table:**

Rigaku AFC12K/SATURN724 diffractometer	1935 independent reflections
Radiation source: fine-focus sealed tube	1811 reflections with *I* > 2σ(*I*)
graphite	*R*_int_ = 0.022
ω scans	θ_max_ = 25.0°, θ_min_ = 3.1°
Absorption correction: multi-scan (*ABSCOR*; Higashi, 1995)	*h* = −5→4
*T*_min_ = 0.759, *T*_max_ = 1.000	*k* = −13→13
2790 measured reflections	*l* = −14→14

### Refinement

**Table:**

Refinement on *F*^2^	Primary atom site location: structure-invariant direct methods
Least-squares matrix: full	Secondary atom site location: difference Fourier map
*R*[*F*^2^ > 2σ(*F*^2^)] = 0.042	Hydrogen site location: inferred from neighbouring sites
*wR*(*F*^2^) = 0.117	H atoms treated by a mixture of independent and constrained refinement
*S* = 1.00	*w* = 1/[σ^2^(*F*_o_^2^) + (0.0744*P*)^2^ + 0.1759*P*] where *P* = (*F*_o_^2^ + 2*F*_c_^2^)/3
1935 reflections	(Δ/σ)_max_ < 0.001
158 parameters	Δρ_max_ = 0.20 e Å^−^^3^
1 restraint	Δρ_min_ = −0.23 e Å^−^^3^

### Special details

**Table:**

Geometry. All s.u.'s (except the s.u. in the dihedral angle between two l.s. planes) are estimated using the full covariance matrix. The cell s.u.'s are taken into account individually in the estimation of s.u.'s in distances, angles and torsion angles; correlations between s.u.'s in cell parameters are only used when they are defined by crystal symmetry. An approximate (isotropic) treatment of cell s.u.'s is used for estimating s.u.'s involving l.s. planes.
Refinement. Refinement of *F*^2^ against ALL reflections. The weighted *R*-factor *wR* and goodness of fit *S* are based on *F*^2^, conventional *R*-factors *R* are based on *F*, with *F* set to zero for negative *F*^2^. The threshold expression of *F*^2^ > 2σ(*F*^2^) is used only for calculating *R*-factors(gt) *etc*. and is not relevant to the choice of reflections for refinement. *R*-factors based on *F*^2^ are statistically about twice as large as those based on *F*, and *R*- factors based on ALL data will be even larger.

### Fractional atomic coordinates and isotropic or equivalent isotropic displacement parameters (Å^2^)

**Table:**

	*x*	*y*	*z*	*U*_iso_*/*U*_eq_	
O1	0.6343 (3)	0.67141 (10)	0.18059 (9)	0.0287 (3)	
H1o	0.584 (5)	0.692 (2)	0.2479 (12)	0.050 (6)*	
O2	0.7598 (3)	0.88248 (10)	0.23009 (9)	0.0315 (3)	
C1	0.7523 (3)	0.77587 (13)	0.16270 (12)	0.0235 (3)	
C2	0.8828 (3)	0.74881 (14)	0.05272 (12)	0.0227 (3)	
C3	1.0455 (3)	0.85068 (14)	0.03269 (13)	0.0278 (4)	
H3	1.0702	0.9349	0.0878	0.033*	
C4	1.1716 (4)	0.82926 (15)	−0.06766 (14)	0.0311 (4)	
H4	1.2846	0.8987	−0.0809	0.037*	
C5	1.1331 (4)	0.70640 (15)	−0.14890 (13)	0.0289 (4)	
H5	1.2192	0.6919	−0.2178	0.035*	
C6	0.9695 (4)	0.60477 (15)	−0.12974 (13)	0.0294 (4)	
H6	0.9428	0.5208	−0.1856	0.035*	
C7	0.8444 (3)	0.62603 (14)	−0.02863 (13)	0.0263 (3)	
H7	0.7326	0.5564	−0.0152	0.032*	
N1	0.4834 (3)	0.73022 (11)	0.39929 (10)	0.0214 (3)	
N2	0.9261 (3)	0.54922 (10)	0.52702 (10)	0.0211 (3)	
C8	0.6169 (3)	0.69350 (12)	0.48783 (12)	0.0190 (3)	
C9	0.6033 (3)	0.75148 (13)	0.60760 (12)	0.0219 (3)	
H9	0.7002	0.7236	0.6683	0.026*	
C10	0.4469 (3)	0.85009 (13)	0.63643 (12)	0.0233 (3)	
H10	0.4365	0.8918	0.7175	0.028*	
C11	0.3052 (3)	0.88738 (13)	0.54546 (13)	0.0231 (3)	
H11	0.1932	0.9541	0.5627	0.028*	
C12	0.3304 (3)	0.82529 (13)	0.42883 (13)	0.0230 (3)	
H12	0.2344	0.8515	0.3668	0.028*	
C13	0.7833 (3)	0.58938 (13)	0.45006 (12)	0.0209 (3)	
H13	0.7846	0.5516	0.3674	0.025*	

### Atomic displacement parameters (Å^2^)

**Table:**

	*U*^11^	*U*^22^	*U*^33^	*U*^12^	*U*^13^	*U*^23^
O1	0.0405 (6)	0.0251 (6)	0.0230 (5)	0.0084 (5)	0.0124 (5)	0.0085 (4)
O2	0.0457 (7)	0.0254 (6)	0.0247 (6)	0.0126 (5)	0.0110 (5)	0.0070 (5)
C1	0.0261 (7)	0.0244 (7)	0.0208 (7)	0.0084 (6)	0.0028 (5)	0.0093 (6)
C2	0.0231 (7)	0.0261 (7)	0.0200 (7)	0.0083 (6)	0.0018 (5)	0.0098 (6)
C3	0.0333 (8)	0.0248 (8)	0.0231 (7)	0.0064 (6)	0.0035 (6)	0.0070 (6)
C4	0.0336 (8)	0.0315 (8)	0.0299 (8)	0.0025 (7)	0.0065 (6)	0.0151 (7)
C5	0.0302 (8)	0.0372 (8)	0.0238 (7)	0.0118 (6)	0.0087 (6)	0.0135 (6)
C6	0.0349 (8)	0.0277 (8)	0.0262 (8)	0.0103 (6)	0.0091 (6)	0.0075 (6)
C7	0.0314 (8)	0.0236 (7)	0.0259 (7)	0.0084 (6)	0.0081 (6)	0.0098 (6)
N1	0.0212 (6)	0.0202 (6)	0.0235 (6)	0.0037 (5)	0.0055 (5)	0.0088 (5)
N2	0.0200 (6)	0.0203 (6)	0.0242 (6)	0.0063 (5)	0.0077 (5)	0.0073 (5)
C8	0.0165 (6)	0.0182 (6)	0.0232 (7)	0.0018 (5)	0.0060 (5)	0.0087 (5)
C9	0.0205 (7)	0.0234 (7)	0.0231 (7)	0.0037 (5)	0.0059 (5)	0.0100 (6)
C10	0.0251 (7)	0.0223 (7)	0.0230 (7)	0.0036 (6)	0.0100 (6)	0.0069 (6)
C11	0.0214 (7)	0.0183 (7)	0.0312 (8)	0.0052 (5)	0.0092 (6)	0.0091 (6)
C12	0.0220 (7)	0.0217 (7)	0.0272 (7)	0.0050 (5)	0.0040 (5)	0.0117 (6)
C13	0.0194 (6)	0.0204 (7)	0.0237 (7)	0.0036 (5)	0.0076 (5)	0.0078 (6)

### Geometric parameters (Å, °)

**Table:**

O1—C1	1.3292 (18)	N1—C12	1.3381 (18)
O1—H1o	0.846 (9)	N1—C8	1.3449 (18)
O2—C1	1.2117 (17)	N2—C13	1.2757 (18)
C1—C2	1.496 (2)	N2—N2^i^	1.408 (2)
C2—C7	1.388 (2)	C8—C9	1.3949 (19)
C2—C3	1.391 (2)	C8—C13	1.4698 (18)
C3—C4	1.384 (2)	C9—C10	1.380 (2)
C3—H3	0.9500	C9—H9	0.9500
C4—C5	1.388 (2)	C10—C11	1.386 (2)
C4—H4	0.9500	C10—H10	0.9500
C5—C6	1.385 (2)	C11—C12	1.385 (2)
C5—H5	0.9500	C11—H11	0.9500
C6—C7	1.390 (2)	C12—H12	0.9500
C6—H6	0.9500	C13—H13	0.9500
C7—H7	0.9500		
			
C1—O1—H1o	109.1 (15)	C6—C7—H7	120.0
O2—C1—O1	123.44 (13)	C12—N1—C8	117.83 (12)
O2—C1—C2	123.29 (14)	C13—N2—N2^i^	111.87 (13)
O1—C1—C2	113.26 (12)	N1—C8—C9	122.45 (12)
C7—C2—C3	119.91 (13)	N1—C8—C13	115.34 (12)
C7—C2—C1	121.78 (14)	C9—C8—C13	122.20 (12)
C3—C2—C1	118.31 (13)	C10—C9—C8	118.85 (13)
C4—C3—C2	119.96 (14)	C10—C9—H9	120.6
C4—C3—H3	120.0	C8—C9—H9	120.6
C2—C3—H3	120.0	C9—C10—C11	119.04 (12)
C3—C4—C5	120.05 (14)	C9—C10—H10	120.5
C3—C4—H4	120.0	C11—C10—H10	120.5
C5—C4—H4	120.0	C10—C11—C12	118.57 (12)
C6—C5—C4	120.19 (14)	C10—C11—H11	120.7
C6—C5—H5	119.9	C12—C11—H11	120.7
C4—C5—H5	119.9	N1—C12—C11	123.25 (13)
C5—C6—C7	119.84 (14)	N1—C12—H12	118.4
C5—C6—H6	120.1	C11—C12—H12	118.4
C7—C6—H6	120.1	N2—C13—C8	120.75 (12)
C2—C7—C6	120.06 (14)	N2—C13—H13	119.6
C2—C7—H7	120.0	C8—C13—H13	119.6
			
O2—C1—C2—C7	−173.81 (14)	C12—N1—C8—C9	−0.65 (19)
O1—C1—C2—C7	7.72 (19)	C12—N1—C8—C13	−179.57 (11)
O2—C1—C2—C3	6.0 (2)	N1—C8—C9—C10	0.1 (2)
O1—C1—C2—C3	−172.47 (12)	C13—C8—C9—C10	178.93 (12)
C7—C2—C3—C4	−0.7 (2)	C8—C9—C10—C11	0.8 (2)
C1—C2—C3—C4	179.52 (13)	C9—C10—C11—C12	−1.0 (2)
C2—C3—C4—C5	0.6 (2)	C8—N1—C12—C11	0.4 (2)
C3—C4—C5—C6	−0.2 (2)	C10—C11—C12—N1	0.5 (2)
C4—C5—C6—C7	−0.2 (2)	N2^i^—N2—C13—C8	−179.13 (12)
C3—C2—C7—C6	0.3 (2)	N1—C8—C13—N2	178.03 (12)
C1—C2—C7—C6	−179.93 (13)	C9—C8—C13—N2	−0.9 (2)
C5—C6—C7—C2	0.2 (2)		

Symmetry codes: (i) −*x*+2, −*y*+1, −*z*+1.

### Hydrogen-bond geometry (Å, °)

**Table:**

*D*—H···*A*	*D*—H	H···*A*	*D*···*A*	*D*—H···*A*
O1—H1o···N1	0.85 (2)	1.88 (2)	2.7269 (16)	177 (2)
C11—H11···O2^ii^	0.95	2.54	3.1811 (19)	125
C12—H12···O2^iii^	0.95	2.59	3.4647 (19)	154

Symmetry codes: (ii) −*x*+1, −*y*+2, −*z*+1; (iii) *x*−1, *y*, *z*.
